# Geroprotectors.org: a new, structured and curated database of current therapeutic interventions in aging and age-related disease

**DOI:** 10.18632/aging.100799

**Published:** 2015-09-02

**Authors:** Alexey Moskalev, Elizaveta Chernyagina, João Pedro de Magalhães, Diogo Barardo, Harikrishnan Thoppil, Mikhail Shaposhnikov, Arie Budovsky, Vadim E. Fraifeld, Andrew Garazha, Vasily Tsvetkov, Evgeny Bronovitsky, Vladislav Bogomolov, Alexei Scerbacov, Oleg Kuryan, Roman Gurinovich, Leslie C. Jellen, Brian Kennedy, Polina Mamoshina, Evgeniya Dobrovolskaya, Alex Aliper, Dmitry Kaminsky, Alex Zhavoronkov

**Affiliations:** ^1^ Laboratory of Molecular Radiobiology and Gerontology, Institute of Biology of Komi Science Center of Ural Branch of Russian Academy of Sciences, Syktyvkar 167982, Russia; ^2^ Moscow Institute of Physics and Technology, Dolgoprudny 141700, Russia; ^3^ Laboratory of postgenomic studies, Engelhardt Institute of Molecular Biology of Russian Academy of Sciences, Moscow 119991, Russia; ^4^ School of Systems Biology, George Mason University, Manassas, VA 20110, USA; ^5^ Integrative Genomics of Ageing Group, Institute of Integrative Biology, University of Liverpool, Liverpool L69 7ZB, UK; ^6^ The Shraga Segal Department of Microbiology, Immunology, and Genetics, Center for Multidisciplinary Research on Aging, Ben-Gurion University of the Negev, Beer Sheva, Israel; ^7^ Insilico Medicine, Inc., ETC, Johns Hopkins University, B301, Baltimore, MD 21218, USA; ^8^ The Research Institute for Translational Medicine, Pirogov Russian National Research Medical University, Moscow 117997, Russia; ^9^ Institute of Physiologically Active Compounds of Russian Academy of Sciences, Chernogolovka 142432, Russia; ^10^ Xpansa, Conzl OU, Tallinn 10616, Estonia; ^11^ Infinity Sciences, Inc, Lewes, DE 19958, USA; ^12^ Genetics, Genomics, and Informatics, University of Tennessee Health Science Center, Memphis, TN 38163, USA; ^13^ Buck Institute for Research on Aging, Novato, CA 94945, USA; ^14^ D. Rogachev Center for Pediatric Hematology, Oncology and Immunology, Moscow, Russia; ^15^ The Biogerontology Research Foundation, London, UK; ^16^ The Judea Regional R&D Center, Carmel, Israel

**Keywords:** growth hormone receptor (GHR), muscle-specific knockout, lifespan, aging, frailty, pathology, body composition

## Abstract

As the level of interest in aging research increases, there is a growing number of geroprotectors, or therapeutic interventions that aim to extend the healthy lifespan and repair or reduce aging-related damage in model organisms and, eventually, in humans. There is a clear need for a manually-curated database of geroprotectors to compile and index their effects on aging and age-related diseases and link these effects to relevant studies and multiple biochemical and drug databases. Here, we introduce the first such resource, Geroprotectors (http://geroprotectors.org). Geroprotectors is a public, rapidly explorable database that catalogs over 250 experiments involving over 200 known or candidate geroprotectors that extend lifespan in model organisms. Each compound has a comprehensive profile complete with biochemistry, mechanisms, and lifespan effects in various model organisms, along with information ranging from chemical structure, side effects, and toxicity to FDA drug status. These are presented in a visually intuitive, efficient framework fit for casual browsing or in-depth research alike. Data are linked to the source studies or databases, providing quick and convenient access to original data. The Geroprotectors database facilitates cross-study, cross-organism, and cross-discipline analysis and saves countless hours of inefficient literature and web searching. Geroprotectors is a one-stop, knowledge-sharing, time-saving resource for researchers seeking healthy aging solutions.

## INTRODUCTION

Aging is a complex biological process involving the progressive breakdown of cellular homeostatic mechanisms and buildup of molecular damage [1-4]. Aging opens the door to disease and a gradual decline in function, and as such anti-aging interventions have been sought since the beginning of time. Modern times call for the expedition of this search. Age demographics are shifting to the right and at record rates [5, 6], suppressing economic growth in developed countries and increasing the risk of economic collapse [7]. With this shift, Alzheimer's disease prevalence will soar in the coming decades [8], as will that of a number of other chronic, debilitating diseases requiring long term health care [9].

Geroprotectors are interventions that aim to prevent, slow, or reverse the processes of aging in model organisms or humans such that the lifespan, and particularly the healthy lifespan, can be extended. They can target any one of many pathways and gene networks involved in aging. While most have only been tested in model organisms, literally hundreds of geroprotectors now exist [10-15]. So many are now under investigation, in fact, that there is a need to catalog and classify these in a centralized repository and to create qualifying criteria for defining geroprotectors. A working model, based on these criteria, would enhance predictive capabilities in screening and ranking potential geroprotective compounds. This, in turn, would facilitate targeted identification and development of new geroprotectors, thus accelerating time to clinic and reducing costs overall [16].

Currently, there are several functional approaches to identifying changes that occur during aging and patterns common to both aging and disease [17, 18]. One approach is to help identify geroprotectors that minimize the pathological changes at various levels of organismal organization. Other approaches may include testing compounds that may delay or prevent the onset of cancer, CNS pathologies, and other age-related diseases [19-21]. There is a clear need for a tracking system that would keep the knowledge of potential geroprotectors accessible and up to date and, in doing so, provide the foundation for evaluating possible geroprotective effects in humans.

A system that could track lifespan experiments and the various properties of each individual geroprotector, such as its side effects, toxicity, and results of prior clinical trials, would serve as a good starting platform for scientists planning longevity experiments or attempting to validate theoretical predictions. To address the growing need for a credible online system for tracking drugs that extend lifespan of various organisms, we have created Geroprotectors.org, a manually curated online database that provides instant access to all of the above. An up-to-date, rapidly explorable system that catalogues and summarizes over 200 geroprotective compounds and links them to over 250 studies that support (or refute) their effects in model organisms, Geroprotectors is for the entrant or expert in the field alike. Its interface is sophisticated, streamlined, and modern, minimizing effort for the user. Its data is manually curated by a team of experts in the field and backed with peer-reviewed literature. Whether browsing or seeking in-depth information on a geroprotector of interest, users can do so without investing time in extensive literature searches or learning complicated systems. Geroprotectors is an intuitive, visual, comprehensive collection of anti-aging interventions that will be a valuable biogerontological resource. Presently, it only tracks drugs that have been shown to extend lifespan in model organisms. However, since some of the drugs that are effective against aging-associated pathologies in humans may also act as geroprotectors [22, 23], these drugs may also be included in the database in the future. These drugs can be submitted using the “submit” form.

## RESULTS

**Figure 1:**
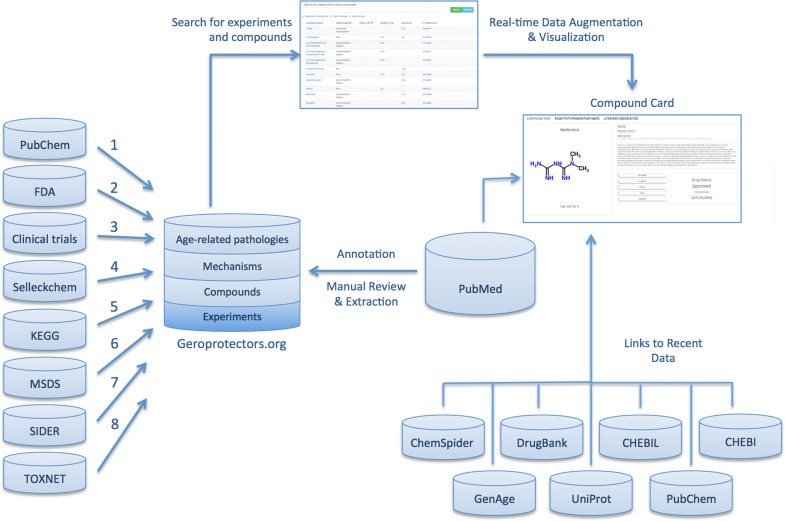
Illustration depicting the content, data sources, and user-directed flow of Geroprotectors.org.

The motivation behind the creation of the Geroprotectors database was to provide a one-stop resource for researchers interested in anti-aging compounds, saving countless hours of data mining, literature review, and expert analysis. As a result, a platform for cross-species, cross-study comparison of the effects of these compounds was created. The interface was developed to make it visually appealing and intuitive for rapid, effortless overviews of geroprotective compounds, with links to original studies and other databases for users seeking further detail. The site was not designed as a mere list of geroprotectors and their phenotypic effects; instead, a comprehensive intervention profile was created for each compound, including its biochemistry and bioactivity, its life-extending effects (and refutations), its toxicity and side effects, and its current drug status. We molded compound profiles around criteria that will be important in developing a working model for geroprotector candidacy. Figure 1 presents a visual overview of the content, data sources, and user-directed exploration of these within Geroprotectors.

### Analysis of experiments related to geroprotective compounds

The database contains summaries of more than 250 experiments involving over 200 geroprotective compounds. Each study was manually selected from the existing biomedical literature by searching the PubMed database (http://www.ncbi.nlm.nih.gov/pubmed) using keywords relevant to pharmacological interventions in aging. Effects on lifespan and experimental conditions (e.g. age, gender, nutrient medium, model organism) were then retrieved. Lifespan parameters included average, median, and maximum lifespan and reduced mortality. These were entered into Geroprotectors in table format, facilitating cross-organism and cross-study comparison of results. All entries in Geroprotectors have links to original publications, making access to raw data fast and convenient. For any given compound, links to relevant studies can be accessed directly from search results or within each compound profile under the Lifespan Experiments tab, which contains a convenient at-a-glance summary of experimental conditions and results. Criteria for inclusion of studies were as follows:

1. Articles contain clear information about model organism, compounds tested, experimental conditions, age at treatment, and results.
2. Experiments were performed on multicellular and/or unicellular organisms. *In vitro* studies were excluded to avoid the question of whether *in vitro* replicative and chronological life extension translates to whole organism life extension.
3. Experiments were performed using wild-type strains to avoid model animals with mutations predisposing them to a particular pathology.

Articles refuting life extending effects of candidate geroprotectors were also included to ensure objectivity and allow users to be informed of and interpret any contradicting findings. These articles are listed and linked under Opposite Effect within each compound's Lifespan Experiments page.

### Analysis of compounds with geroprotective activity

In addition to information about lifespan experiments for each compound, we provide biochemical and mechanistic profiles for each entry that are comprehensive yet presented in an at-a-glance style for user convenience. We developed the profiles in accordance with what we wish to promote as criteria important in developing a working model of geroprotectors. Compound profiles included the following:

1. *Aliases*: For a convenient and effective search, we included aliases of substances tested in experiments. This data was obtained from PubChem database (http://www.pubchem.ncbi.nlm.nih.gov).
2. *FDA Drug Status*: Several federal drug administration (FDA)-approved compounds with acceptable safety profiles in humans were shown to have geroprotective effects in model organisms. An FDA/Center for Drug Evaluation and Research database (http://www.accessdata.fda.gov/scripts/cder/drugsatfda/index.cfm) search was performed using Drug Name, Active Ingredient, or Application Number to find out whether each compound is FDA-approved. The status of each compound was gained from DrugBank Therapeutic Target Database, which uses the following notation:
  - *Not a drug* - not used as medical drug
  - *Experimental* - experimental drug, routine usage in medicine and veterinary applications, not yet permitted by national regulatory authority
  - *Approved* - approved for use as drug
  - *Withdrawn* - withdrawn because of possible risk to patients with unexpected side effects, which were not detected during phase III clinical trials
  - *Investigational* (in the USA) - approved for use by national regulatory authority in special cases (usually in key clinical trials) to enter it into the market while monitoring the safety of the drug
  - *Clinical Trials* - drug in stage of clinical trials
3. *Number of clinical trials*: Information retrieved from clinicaltrials.gov. Currently, this is the largest web-catalog of registered clinical trials. Launched by the United States National Library of Medicine, it contains information about more than 192,862 clinical trials conducted in the US and 189 other countries, including comprehensive descriptions of protocols, conditions, studied drugs, etc. Statuses of some compounds were formed on the basis of information in the literature.
4. *Toxicity*: Since acceptable toxicity is one of the main criteria for geroprotector, we also added information concerning the toxicity of each substance as evaluated by oral LD_50_ in three model organisms (mouse, rat, and rabbit). Oral LD_50_ values were collected from material safety data sheets (MSDS) provided by suppliers. Toxicology data were also obtained from the database http://toxnet.nlm.nih.gov/.
5. *Targets*: Target-based and systems-based approaches for aging therapy are showing great promise. From this perspective, we included targets activated and inhibited under the influence of candidates to geroprotectors. The data was obtained from KEGG.
6. *Side Effects*: Geroprotectors should have little or no side effects and no adverse effects. Therefore, we carried out a PubMed search and listed the databases below to reveal mentioned effects:
  http://sideeffects.embl.de/drugs/5040/
  http://www.fda.gov/Safety/MedWatch/SafetyInformation/default.htm
  http://toxnet.nlm.nih.gov/
  http://www.reference.md/
  http://livertox.nih.gov/
7. *Microbes*: Microbes may preserve host function through regulation of energy homeostasis and immunity during reproductive life; however, it is also conceivable that selection occurs for organisms that contribute to host demise after reproductive life [24]. Therefore we implemented an additional search both of PubMed and http://www.selleckchem.com/ to find out whether candidates to geroprotectors possess antibacterial, antifungal or antiviral activity.
8. *Age-related pathologies and mechanisms*: We also investigated associations between compounds and age-related pathologies (chronic inflammation, cancer, amyloid aggregation etc.), activation of pro-longevity mechanisms (hormesis, caloric restriction mimetics, stress resistance etc.), and suppression of pro-aging mechanisms (metal ion chelating, antioxidant defense, excessive protein biosynthesis inhibition, cellular senescence). Each of these is an important factor in rating potential geroprotectors. Age-related pathologies were selected based on the availability of data about their connection with compounds in the database.
  Mechanisms of aging and longevity were selected based on major theories of aging, such as the theory of mutation accumulation, the theory of programmed cell death, etc. [4].
9. *Biomarkers of aging*: Biomarkers of aging are minimally or non-invasive, universal and stable physiological and biochemical indicators which reflect real biological age [25]. They are unique, easily detectable and measurable factors, which can change over an individual's lifespan [26, 27]. Therefore, it is particularly important in screening for compounds which have potential geroprotective effects to understand their influence on the biomarkers of aging. For this reason, we have identified studies that demonstrate the influence of the compounds annotated in our database on aging biomarkers.
10. *Other links*: Databases additionally contain links to Gene Expression Omnibus (www.ncbi.nlm.nih.gov/geo/), transcriptional signature changes from LINCSCLOUD L1000 assay (www.lincscloud.org/), natural sources of substances that are not synthetized, and structural analogs. All databases integrated with Geroprotectors.org are summarized in Table 1.

**Table 1:** Summary of biological, chemical, and drug databases integrated within Geroprotectors.org

Information source	Link	Description
Chemical databases		
PubChem [28,29]	https://pubchem.ncbi.nlm.nih.gov/	One of the largest chemical databases on the web. Created and maintained under the National Center for Biotechnology Information (NCBI). Contains over 68 million entries for compounds and over 198 million entries for substances, including mixtures, complexes, uncharacterized substances, etc. Each entry has a full description of the chemical and biological properties, utilization of the substance, information about vendors, etc.
Chemical Entities of Biological Interest (ChEBI) [30-32]	https://www.ebi.ac.uk/chebi/	Free access chemical database focused mainly on small molecules. Every entry provides information about names, synonyms, registry number(s), molecular formula, and main chemical descriptors. This database is a part of the European molecular biology laboratory (EMBL) project.
ChEMBL [33-35]	https://www.ebi.ac.uk/chembl/	Similar to ChEBI, but focused on compounds with drug-like or potential drug properties; contains over 1.7 million entries. Highlights the advantages of advanced search instruments. Search by ligand structure, current compound targets, and other keywords available on the web. Accepted by ELMB.
ChemSpider [36-38]	http://www.chemspider.com/	Integrative chemical database from Royal Society of Chemistry. Includes 43 million chemical structures from 490 data sources. Provides information about patents, vendors, etc., related to each annotated compound.
Drugs databases		
DrugBank [39-41]	http://www.drugbank.ca	Comprehensive resource indexing drugs at any stage—approved for human use, in clinical trials, and experimental drugs. Summarizes each drug's influence on molecular targets. Also provides information about pharmacology, side effects, etc.
The Pharmacogenomics Knowledgebase (PharmGKB) [43]	https://www.pharmgkb.org/	Shows interactions between drugs and genes.
Side Effects Data Base [44]	http://sideeffects.embl.de/	Describes over 4,000 side effects (adverse events) of 996 approved drugs.
Therapeutic Targets Database (TTD) [45,46]	http://bidd.nus.edu.sg/group/cjttd	Provides information about cell therapeutic targets, related metabolic pathways, and corresponding drugs. Provided by National University of Singapore.
Metabolic pathways databases		
Kyoto Encyclopedia of Genes and Genomes (KEGG) [47]	http://www.genome.jp/kegg/	Resource including various databases such as chemical, biological, metabolic pathways, drug, and disease databases. Developed in Kyoto University.
The Human Metabolome Database (HMDB) [48,49]	http://www.hmdb.ca/	Database of metabolites detectable in the human body. First resource designed for metabolomics; now contains over 42,000 entries.
Toxicology databases		
TOXNET [50]	http://toxnet.nlm.nih.gov	Contains a variety of resources related to toxicology, linked to original peer-reviewed articles.
Liver Tox [51]	http://livertox.nih.gov/	Provides data on hundreds of compounds with known or potential liver toxicity.
Gene Expression		
Gene Expression Omnibus [52,53]	http://www.ncbi.nlm.nih.gov/geo	International public repository archiving microarray, next generation sequencing, and other genomics data; user submitted.
L1000 [54]	http://www.broadinstitute.org/LINCS	Project of the Broad Institute. Utilizes computationally selected 1000 landmark genes to infer the transcriptome.
Protein databases		
UniProt [55]	http://www.uniprot.org/	The largest database of the annotated proteins.
Clinical trials databases		
Clinical Trials [56,57]	https://clinicaltrials.gov/	The largest web catalog of registered clinical trials launched by the US National Laboratory of Medicine; contains over 200 clinical trials conducted in US and nearly 200 more conducted abroad. Describes protocols, conditions, studied drugs, etc.
Other sources		
MeSH (Medical Subject Headings)	https://www.nlm.nih.gov/mesh/	US National Library of Medicine's controlled vocabulary thesaurus.
Reference.MD	http://www.reference.md/	Resource that integrates medical information from MeSH, Drugs@FDA, FDA Adverse Event Reporting System, etc.
Commercial organizations (Chemical vendors)		
Chemnet	http://www.chemnet.com/cas/	China-based company; platform of providing comprehensive service to chemicals; database of 300,000 products.
Sigma Aldrich	http://www.sigmaaldrich.com/	US-based company; manufactures over 230,000 chemicals, biochemicals, and other essential products; 1.4 million customers globally.
Enzo Life Sciences	http://www.enzolifesciences.com/	US-based manufacturer of health and biological sciences research products.
Santa Cruz Biotechnology, Inc.	http://www.scbt.com/	US-based developer of products for biomedical research worldwide.

### Visualization and data structure of database

The Geroprotectors database integrates information submitted in four blocks (Figure 1):

1. Lifespan experiments: abstracts and summarized results of studies demonstrating life-extending effects of a given compound.
2. Compounds: data about geroprotective compounds.
3. Mechanisms: involvement of compound in well-known mechanisms of longevity and aging suppression.
4. Age-related pathologies: actions of compound on the most prevalent and well-studied age-related conditions and associated diseases.

The compounds data is divided into 3 blocks. The first block contains image, name, a short description, and links to other chemical databases. It also includes information about the number of clinical trials and drug status. The second block includes biological, pharma-cological, toxicological information and mechanisms of longevity. The third block is dedicated to each compound's associations with age-related pathologies (chronic inflammation, cancer, amyloid aggregation etc.).

### Integration with biochemical and drug databases

Compounds and mechanisms were described using multiple chemical and biological databases. Compound profiles include a short description, any aliases, chemical structure, toxicity profile, clinical usage, biological and pharmacological activity, chemical interactions, etc. All substances were integrated with the top chemical databases, including PubChem, ChemSpider, DrugBank, CHEBIL, UniProt, and GenAge (Table 2).

**Table 2:** Summary of chemical databases linked to compound profiles within Geroprotectors.org

Data Base	Link to the resource	Number of compounds presented in current database
PubChem^28, 29^	https://pubchem.ncbi.nlm.nih.gov/	209
ChemSpider^36-38^	http://www.chemspider.com/	208
ChEMBL^33-35^	https://www.ebi.ac.uk/chembl/	193
ChEBI^30-32^	https://www.ebi.ac.uk/chebi/	158
DrugBank^39-42^	http://www.drugbank.ca/	98

**Figure 2:**
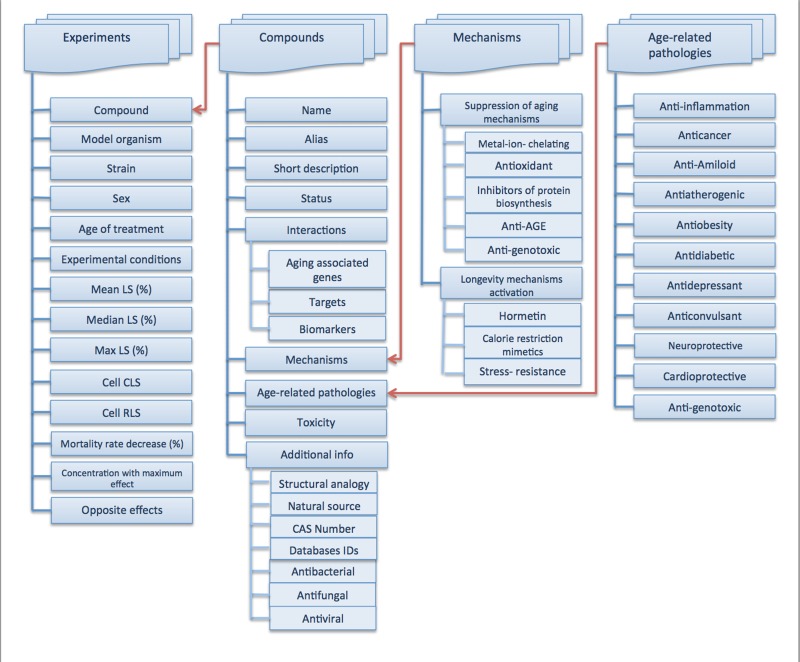
Data structure of Geroprotectors.org, showing that data is structured into 4 main blocks: Experiments, Compounds, Mechanisms, and Age-Related Pathologies, each of which contains multiple blocks of information providing a well-rounded profile of geroprotective compounds under investigation.

### Comparison with existing aging databases

Several free-access databases already exist in regards to aging research, each of which fills a specific need and takes a unique approach (Table 3). Some of these databases do contain information about geroprotectors, particularly AgeFactDB by JenAge, which profiles phenotypic effects of 91 compounds associated with aging, along with genes and other interventions related to aging. However, none provides a comprehensive list of current and candidate geroprotectors and complete summary of information on each compound. Also, none contain biochemical and mechanistic profiles of the geroprotective compounds integrated with external databases. At its launch, Geroprotectors contains more than double the amount of geroprotectors than any other aging database, with more information provided per compound, and with continued manual curation to ensure that this information remains current.

**Table 3:** Summary of existing aging-related databases for comparison with Geroprotectors.org

Source	Info structure	Organisms associated with experiment	Number of gero-protectors in database	Life span effects of compound	Compound structure and toxicity info	Compound relation to biomarkers of aging
AgeFactDB by Jen Age^58^ (http://www.agefactdb.jenage.de/)	tables	17	91	yes	no	yes
Human Ageing Genomic Resources^59^ (http://genomics.senescence.info/)	multiple databases; varied	1-4000+ depending on database	0; focus is on genes, not compounds	no	no	no
Lifespan Observation database by Sageweb (http://lifespandb.sageweb.org/)	tables	17	91	yes	no	no
Geroprotectors (http://www.geroprotectors.org/)	tables	17	256	yes	yes	yes

### Use Case

A researcher who works with *C. elegans* wants to investigate the known geroprotective effects of the drug Resveratrol and visits geroprotectors.org to gain information that will aid design of an experiment. On the home page, he searches for Resveratrol. The search retrieves seven references to experiments that demonstrate geroprotective effects of Resveratrol, performed in four model organisms [60-66]. These references are presented in simple table format, with one row for each study cited. Columns include the mean, median, and max lifespan extension percentages from each study and PubMed reference links. Using median lifespan for example, the researcher can instantly see that Resveratrol has been shown to increase this measure by 10-17.8% in *C. elegans* [62, 63, 66], 56% in *Nothobranchius furzeri* [65], and 13% in *Drosophila melanogaster* [64]. At this point, the researcher can do one of two things: 1) go directly to the literature to read more about any one of these seven studies by clicking on the corresponding PubMed links in the last column of the table, or 2) explore the properties of Resveratrol and more about these studies within geroprotectors.org.

Seeking a rapid, broad overview of the drug and further information about the increase in *C. elegans* lifespan, this user chooses the latter, to stay within the Geroprotectors database. A click on the Resveratrol link in any row of the table leads to the summary page for Resveratrol. This includes an image of the chemical structure of Resveratrol and tabs above this image that allow selection of one of three summary profiles of this drug: Compound Info, Bioactivity/Pharm/Pathways, or LS Experiments (LS referring to lifespan).

Since Compound Info is the current tab (with the image of Resveratrol structure), the researcher begins here. Scrolling down beneath the image, the drug name and aliases, Wikipedia entry for the drug, and links to chemical and drug databases (Chemspider, DrugBank, ChEMBL, ChEBI, PubChem) appear. Finally, at the bottom of this page, the current drug status for Resveratrol appears (experimental, investigational), as does the number of clinical studies performed using this drug (95 in this case). To immediately access these, the researcher can follow the link to clinicaltrials.org.

The second tab, Bioactivity/Pharm/Pathways, helps the researcher to learn more about biological activity of Resveratrol. Instantly on the left, a list of over 30 interactions with Resveratrol, compiled from and linked to UniProt, appears. On the right, the researcher can see that Resveratrol has several types of effects on an organism, including anti-inflammation, anti-cancer, anti-atherogenic, anti-obesity, neuroprotective, and cardioprotective effects. Links to the studies that support each of these effects are provided here. Below the organism effects list is a quick yes/no summary of the antibacterial, antifungal, and antiviral properties of a drug, and the researcher sees that Resveratrol has none of these. Scrolling down, there is also a list of longevity mechanisms of action for Resveratrol, which include calorie restriction mimetics and stress resistance, and suppression of aging mechanisms, which for Resveratrol include antioxidant properties. Finally, a statement at the bottom of this page indicates whether or not Resveratrol is related to biomarkers of aging (it is not), and at the bottom left of this page, the researcher can find related pathways and LD_50_ levels that index toxicity.

If the researcher still wants to learn more about the experiments extending lifespan with Resveratrol, the LS Experiments Tab can provide more information. Exploring this tab, on the left, the title and abstract of one of the seven articles originally listed in the table following the homepage search appears. This abstract is for an experiment with Saccharomyces cerevisiae [61]. This and the other six articles are accessed by tabs at the top of this page. To access the C. elegans experiments, the researcher clicks on one of the two C. elegans tabs and the title abstract for one of the three studies appears. Below the abstract, the researcher can see that strain N2 was used. To the right, he sees that the experimental conditions involved agar nematode growth medium + E. coli OP50, and that median life extension for this study was 17.8% [62]. He also sees that 1mM was the concentration of Resveratrol with maximum effect in this study. Scrolling down, there is an additional link to this study followed by a link to a study with opposite effect.

### Availability

The Geroprotectors database is available at http://www.geroprotectors.org with the data made available under the permissive Creative Commons licence, allowing data to be used in other analyses. There are options to either download the entire database or to download more focused data using the export tool. Feedback via email is welcome, as are submissions of new data, for which a submission form is provided to ensure that the relevant information is included.

## DISCUSSION

Geroprotectors.org is a unique database publicly available online. Other age-related databases are generally tailored to basic knowledge of longevity/mortality genes, genomes of long-living species (naked mole-rat, bowhead whale, etc.), and aging theories, with relatively little information about anti-aging interventions. Geroprotectors.org provides information about life-extending effects of more than 230 geroprotective compounds and summarizes other key data about these compounds in a one-stop, rapidly explorable, clean, sophisticated and intuitive interface. The database is backed by over 250 peer-reviewed studies and chemical/drug databases, which are summarized and linked throughout the site, ensuring literature-supported data, quick and convenient access to original publications, and due credit to the authors of the works. In addition to efforts to ensure accuracy in manual curation, every effort was also made to include studies that refute geroprotective effects to maintain objectivity and ensure a comprehensive look at each compound. As aging continues to rise to the forefront of the list of social and economic problems in the coming decades, resources such as Geroprotectors that enable the multidisciplinary community of biogerontologists and clinicians to come together and share knowledge will become increasingly crucial. In response to this demand, Geroprotectors provides a valuable knowledge-sharing resource for researchers interested in anti-aging solutions.
